# The Radiation Response of Sarcomas by Histologic Subtypes: A Review With Special Emphasis Given to Results Achieved With Razoxane

**DOI:** 10.1155/SRCM/2006/87367

**Published:** 2006-05-11

**Authors:** Walter Rhomberg

**Affiliations:** Department of Radiooncology, General Hospital, Carinagasse 47, 6800 Feldkirch, Austria

## Abstract

*Purpose*. Relatively few results are available in the literature about the radiation response of unresectable sarcomas in relation to their histology. Therefore, an attempt was made to summarize the present situation. *Materials and methods*. This report is based on a review of the literature and the author's own experience. Adult-type soft tissue sarcomas, chondrosarcomas, and chordomas were analyzed. Radioresponse was mainly associated with the degree of tumor shrinkage, that is, objective responses. Histopathologic responses, that is, the degree of necrosis, are only discussed in relation to radiation treatment reports of soft tissue sarcomas as a group. *Results*. Radiation therapy alone leads to major responses in about 50% of lipo-, fibro-, leiomyo-, or chondrosarcomas. The response rate is less than 50% in malignant fibrous histiocytomas, synovial, neurogenic, and other rare soft tissue sarcomas. The response rates may increase up to 75% through the addition of radiosensitizers such as halogenated pyrimidines or razoxane, or by the use of high-LET irradiation. Angiosarcomas become clearly more responsive if biologicals, angiomodulating, and/or tubulin affinic substances are given together with radiation therapy. Razoxane is able to increase the duration and quality of responses even in difficult-to-treat tumors like chondrosarcomas or chordomas. *Conclusions*. The available data demonstrate that the radioresponsiveness of sarcomas is very variable and dependent on histology, kind of radiation, and various concomitantly given drugs. The rate of complete sustained remissions by radiation therapy alone or in combination with drugs is still far from satisfactory although progress has been made through the use of sensitizing agents.

## INTRODUCTION

Little is known about the quality and duration of objective responses in different sarcomas after definitive or palliative radiation therapy. The few reports on photon irradiation deal mainly with soft tissue sarcomas (STS) as a group [1, 2]. Radiation therapy alone leads to transient objective response rates up to 50% and local tumor control in about 30% of unresectable lesions with radiation doses in the order of 60 to 70 Gy [1–3]. Improvements in local control and response rates between 50% and 75% have been achieved by high-LET irradiation [4–7], the use of radiosensitizers such as intravenous bromodeoxyuridine, iododeoxyuridine [8], razoxane [9], or hyperthermia. Slater et al analyzed 72 patients with unresectable STS and found a relationship between local control rates and so-called malignancy groups based on pathologic diagnosis [1]. The aim of the present paper is to continue that analysis of the radioresponsiveness of the main histologic groups of sarcomas.

## MATERIALS AND METHODS

This review is based on the available literature and on the author's own institutional experience gained during studies of radiosensitizing agents in the treatment of sarcomas from 1972 to 2004. The responses to irradiation of unresectable primaries, recurrent and measurable metastatic lesions from adult-type STS, chondrosarcomas, and chordomas were analyzed; those from desmoids, rhabdomyosarcomas, osteogenic sarcomas, and Kaposi sarcoma were excluded.

Our radiation treatment was performed with high energy photons using shrinking field techniques. Most patients received 180–200 cGy per fraction, usually five times a week. The median total tumor dose was 58 Gy (range: 30–70 Gy) for the most frequent STS, 60 Gy (range: 54–65 Gy) for chondrosarcomas, and 63 Gy (range: 54–67 Gy) for chordomas. The sensitizer razoxane was given by mouth at a dose of 125 mg twice daily to selected patients according to study protocols. Drug treatment commenced 5 days before the first irradiation treatment and was continued on radiation days.

### Definition of radiation response

Response to irradiation has to be seen from two aspects. Sarcomas may be radioresponsive or even radiocurable in terms of cell killing but not responsive in terms of shrinking of the mass [10, 11]. Brennan et al wrote, “this can be perplexing, as such masses could give the false impression that little has been accomplished, whereas in reality the mass is mainly made up of sterilized tumor cells and debris” [10]. The degree of tumor cell sterilization (necrosis) may be of value with respect of local control and survival [12]. However, the assessment of the degree of necrosis necessitates a histopathologic investigation and is, therefore, mostly restricted to neoadjuvant preoperative studies. Moreover, considerable necrosis may preexist in many sarcomas before any treatment has been given. To be clinically useful, the degree of pathologic necrosis should reach some 95%. This value was found to be an independent prognostic factor for local control and survival [12]. Willett et al found that moderate dose preoperative radiotherapy resulted in 80% necrosis in 21 of 27 specimens. Grade and size (> 10 versus < 10 cm) were important predictors of response to radiotherapy independent of histologic type [13].

In this review, radiation response will in the main be related to the extent of objective tumor shrinkage defined according to standard criteria. A complete response (CR) required the complete disappearance of a tumor both clinically and by radiographic imaging. A partial regression (PR) meant a reduction of the initial tumor volume by more than 50%, and disease progression (P) was assumed if the pretreatment tumor volume increased over 25% during or shortly after the end of the radiotherapy. “No change” (NC) described a response between PR and progression. Local tumor control was defined as freedom from symptoms and no regrowth of the tumor at the site of irradiation as long as the patient survived.

Definitions of tumor responsiveness are often a matter of debate. A proposal could be as follows: a sarcoma type is regarded as radioresponsive if the majority of tumors show an objective regression with a CR rate up to 25% and proven survival benefits due to adjuvant irradiation. Sarcomas were designated moderately responsive if less than 50% of the tumors reduced in size in terms of a partial and occasional complete regression. Resistant sarcomas are associated with a major objective response rate below 25%, and a majority of them possibly exhibit progressive disease within 3 months after the start of radiotherapy.

## RADIATION RESPONSE BY HISTOLOGIC SUBTYPE

### Fibrosarcomas

In the past, this subtype of STS was more frequently diagnosed than today since it probably included some cases of malignant fibrous histiocytoma or gastrointestinal stromal tumors. There is controversy about the radioresponses of these tumors. Fibrosarcomas were largely regarded as radioresistant, especially the subunit of dermatofibrosarcoma protuberans. This, however, was recently questioned at least for the adjuvant setting [14]. In addition, almost 40 years ago, Windeyer et al described a satisfactory local control by radiotherapy in the adjuvant setting and an objective response rate of 50% even in inoperable, recurrent, or residual fibrosarcomas [15].

Fibrosarcomas were one of the first entities that were subjected to irradiation together with the radiosensitizer razoxane (ICRF 159) in England. Ryall et al reported an objective response rate of 73% of STS by radiotherapy combined with razoxane. The majority of their cases were fibrosarcomas [16]. We found an overall response rate of 70% in a pilot series of 10 patients with fibrosarcomas treated with radiotherapy and razoxane between 1973 and 1977. The median response duration was 8 months. But later, among 8 chemonaive patients with fibrosarcomas subjected to irradiation together with razoxane, there were 5 CR and 3 PR, and the median duration of response rose to 15 months emphasizing the influence of the pretreatment. Several times a striking liquefaction of the tumors could be noted clinically. Subgroup analysis within a randomized study [9] showed that 6 of 7 patients with a fibrosarcoma subjected to irradiation together with razoxane achieved an objective response (3 CR and 3 PR, resp), confirming the results of our pilot study and the early results of Ryall et al [16]. Thus, the rate of major responses in our 17 patients with measurable fibrosarcomas subjected to irradiation together with razoxane was 76%. For comparison, among 5 measurable cases randomized to be treated with radiotherapy alone, we saw 2 PR and 3 unchanged tumors. Taken together, there seems to be a clear improvement of the radiation response rate in the group of fibrosarcomas also treated with the radiosensitizer razoxane. Data on a doxorubicin-based chemo-radiotherapy are not available.

### Liposarcomas

Liposarcomas are known to belong to the more sensitive varieties of STS but data on quantitative measurements of remissions are sparse. One of the few studies that provided objective data of the radiation response of myxoid liposarcoma (MLS) was recently published by Pitson et al [17]. In a series of 16 MLS tumor specimens the mean pretreatment and post-treatment volume of the MLS tumors was 415 and 199 cm^3^ corresponding to a reduction in the median tumor volume of 59%. This result achieved by radiotherapy alone was in sharp contrast to 16 control cases of malignant fibrous histiocytomas. In our own experience, among 9 patients with liposarcomas treated with radiotherapy alone, there were 2 CR, 2 PR, 2 NC, and 2 progressive diseases, respectively, and one patient was not assessable. The objective response rate of 50% corresponds approximately to that of the MLS series of Pitson et al [17].

**Figure 1:**
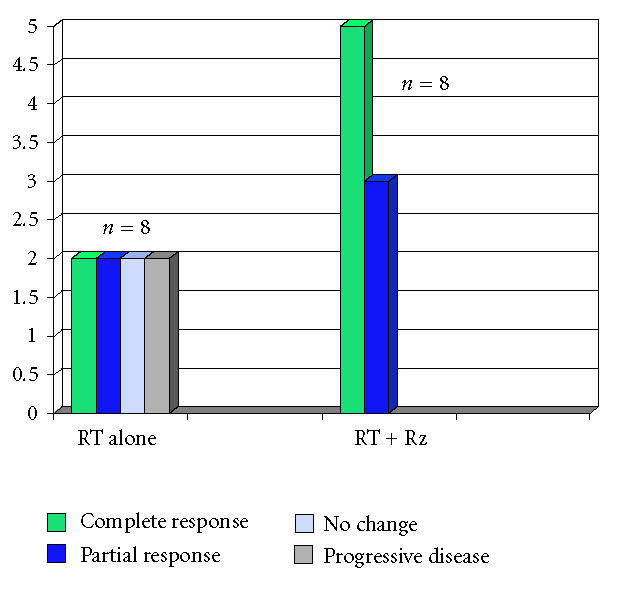
Response of 16 assessable liposarcomas randomized between radiotherapy (RT) alone and together with razoxane (Rz).

These 9 patients were part of a randomized study published in [9]. In that study, another 9 patients were randomized to receive razoxane together with radiation treatment. The median radiation dose at the tumor site was 55 Gy (range: 40–60 Gy). Five of 8 assessable patients had a complete response (CR), and 3 had a partial response (PR) corresponding to a near 100% response rate. One patient was not assessable because of a gross residual unmeasurable tumor. Figure 1 shows a late subgroup analysis of 16 assessable liposarcomas treated within that randomized study. The median follow-up time of the 16 patients (including the patients who died) was 29 months (range: 1.5–110+ months).

### Leiomyosarcomas

Leiomyosarcomas (LMS) occur frequently in the uterus, bowel, vascular tissues, and less commonly in somatic soft tissue or bone. LMS was proven to be the most frequent secondary tumor induced by radiation. Apart from case reports, there are no large series on the radioresponsiveness of LMS. Treatment results are usually restricted to the adjuvant setting. With the exception of LMS of the uterus [18, 19] there is agreement about the necessity and the benefit of postoperative radiation therapy in LMS. In sarcomas of the uterus, LMS were usually compared to other entities like endodermal stromal sarcomas (ESS) and mixed mesodermal sarcomas (MMS). The definitive radiotherapy of uterine leiomyosarcomas is not rewarding. Based on a review of the literature, none of 10 cases of stage II-IV LMS survived 5 years; the same was true also for the MMS and ESS varieties [19]. By combining external beam radiotherapy and brachytherapy, and total doses of 80 Gy, 2 CR of ESS by irradiation alone were observed by Weitmann et al [20]. Perhaps, LMS of the prostate may represent a more radioresponsive entity where cures are possible in the odd case [21, 22].

We treated 9 patients with measurable LMS (primaries and metastases). Among 4 cases treated with radiotherapy alone, 2 PR, 1 NC, and 1 progression were seen leading to an objective response rate of 50%. The duration of the partial responses was 3 and 15 months. When razoxane was added to the radiation treatment in 5 patients, 2 CR and 3 PR with a median duration of 22 months was observed, although one female received salvage surgery. Of course, the small numbers of patients preclude firm conclusions, but the tendency to improve the radiation response by the combination with razoxane is seen also in this type of STS.

### Malignant fibrous histiocytomas

Malignant fibrous histiocytoma (MFH) is a pleomorphic neoplastic disease with uncertain histological origin. Patients have an absolute three-year survival rate between 43% and 72% [23, 24]. The prognosis is strongly dependent on tumor size and the depth of a muscular tumor [24, 25]. Complete surgical resection at the time of primary tumor presentation is the treatment of choice, although adjuvant radiation therapy plays an important role in achieving better local control [25–27]. Responses to primary single and combination chemotherapy occur in about 30% of the patients [24]. Most authors feel that radiotherapy alone or chemotherapy is not effective in MFH.

A literature survey in 1981 revealed that local control was obtained in only 2 of 16 patients treated by radiotherapy alone, but measurement of tumor response or details of the radiation treatment were not well documented [27]. A study of 16 patients with measurement of the radiation response in malignant fibrous histiocytomas came from the Princess Margaret Hospital in Toronto. Fourteen tumors were grade III-IV, the median time from the pre-RT image to the start of treatment was 25 days (range: 4–50 days). All patients received 50 Gy in 2 Gy fractions. Tumor necrosis was quantified in the pathology report after surgery in 10 MFH tumors. A median of 80% of the MFH tumors was necrotic. The mean pretreatment and post-treatment volume of the 16 MFH tumors was 264 and 273 cm^3^, respectively (P = .804) [17]. Thus, the radioresponsiveness of MFH tumors was clearly less than in 16 comparable cases with myxoid liposarcomas from the same institution although the observed degree of necrosis was not different [17].

Among 4 of our cases treated with radiotherapy alone, there were 1 complete response, 1 partial response, and 2 unchanged tumors. Another 4 patients with measurable tumors received irradiation with razoxane, resulting in 2 PR and 2 NC with a median duration of 10.5 months. Two further cases treated with razoxane had incomplete resections of masses that were not measurable, but they remained locally controlled. Summarizing the available data, the radioresponsiveness of MFH in terms of tumor shrinkage seems to be limited. The value of the radiosensitizing agent razoxane remains unclear.

### Synovial sarcomas

This entity accounts for approximately 8% of all STS, and it typically affects the extremities. The translocation (X;18) has been noted in more than 90% of cases. Synovial sarcomas (SS) have a higher level of chemosensitivity compared with other STS [28]. They appear to be particularly sensitive to high-dose ifosfamide chemotherapy [29].

In 1971, Kagan et al observed different responses (CR, PR, and NC) in one patient with multiple metastases of an SS, dependent on the time/dose/fractionation scheme [30]. An early review from Carson et al in 1981 indicates the existence of only a few reports on the use of radiotherapy alone or in conjunction with excision [31]. The authors reported on their own 36 patients with SS, and underlined the unfavorable outcome as soon as the patients developed infiltrative tumors of T-3 stage, or if the tumors showed poorly differentiated histologies. Among 3 macroscopic incomplete resections in primary SS which were irradiated with 40, 58, and 82 Gy, respectively, 2 died of local recurrence after 6 and 24 months, and 1 died from lung metastasis after 6 months, the primary being controlled. Measurement of the response was not performed.

Twenty years later, the question as to objective response rates following irradiation is still not solved but meanwhile there is a consensus on the need for radiotherapy following marginal resection. A large series of 271 synovial sarcomas was recently analyzed by Ferrari et al [28]. The authors found 10 cases which remained unresectable after some pretreatment (mostly with chemotherapy), with radiotherapy being the only local treatment thereafter. Only 1 of 10 survived; response data were not given.

Our own experience comprises 7 patients. Three patients received radiotherapy alone: there were 1 histopathologically confirmed complete response (bone lesion 5 × 5 cm; 60 Gy), 1 partial response (primary at the knee; 55 Gy), and 1 progressive disease at an R-2 resected primary. The patients survived 60, 18, and 12 months, respectively. Four patients were treated with radiotherapy and razoxane by randomization with 1 PR and 3 NC. However, 2 of the unchanged lesions were metastases that received only 30 and 36 Gy.

### Sarcomas of the blood vessels

As a rule, *angiosarcomas* (AS) have a worse outcome in comparison to other STS. They usually present high-grade histology and have a high propensity for local recurrence and distant metastases. However, biology and prognosis are also dependent on the site of origin. Thus, tumors originating on the scalp, face, or the thyroid imply a dismal prognosis compared with those in other locations [32–35]. The 5-year survival is between 0 and 13% especially among patients who were irradiated with clinically evident disease [32, 33, 36, 37]. Present studies, however, indicate a 5-year survival of 45%–60% in all angiosarcoma patients treated with curative intent [32, 37].

Most frequently, radiotherapy is given as an adjunct to surgery in the setting of minimal residual disease. Few data exist about primary or definitive radiotherapy of AS. Single-case reports repeatedly describe complete durable responses with radiotherapy alone [38, 39] but the true incidence of such an outcome remains unknown, and it is probably lower than case reports suggest. In a study by Mark et al [40], only 1 of 9 patients (11%) treated with RT + / − CT was rendered free of disease. We treated 2 patients with radiotherapy alone (by randomization): one patient with a recurrent angiosarcoma of the thyroid measuring 7.5 × 4 cm had progressive disease despite a dose of 60 Gy given in 30 fractions. A partial response was seen in a metastatic lesion on the forehead from an AS of the breast irradiated with 14 Gy only.

Angiosarcomas emerge as chemoresponsive tumors [41–43]. Radiation with innovative drug regimens, for example, cytokines, antiangiogenic substances, and others, seems to become a promising treatment option [44–46]. Ohguri et al used rIL-2 immunotherapy together with radiotherapy (median 70 Gy, single fractions 2–3 Gy) in 20 patients with angiosarcomas of the scalp, 15 of them had no surgery. The median overall survival time was 36 months; the median local recurrence-free survival 11 months. Local recurrences were observed in 7 patients only (35%) [44]. These results were more favorable than any previous report in the reviewed literature [44]. Unfortunately, objective response data are lacking in the article.

We used radiotherapy together with razoxane in 3 patients with unresectable AS on the scalp, the heart, and the lung, respectively. The radiation doses were 66, 30, and 40 Gy. All 3 patients responded partially, 2 with a subtotal regression of their primaries. Distant metastases were the limiting event in all 3 cases associated with survival times of 15, 9, and 8 months, respectively.

In an ongoing study of the Austrian Society of Radiooncology (ÖGRO), using radiotherapy in combination with razoxane and vindesine, a tubulin affinic antiinvasive substance [47], 3 of 7 patients with measurable angiosarcomas showed a CR, 3 had a partial response, and 1 did not change (minor response). The complete responses occurred at unfavorable sites like the thyroid, chest wall, and scalp. No recurrences were seen in the responding patients after a median follow-up time of 17+ months for the living patients (Wink et al, preliminary observations).

The value of radiotherapy in the management of *hemangiopericytomas* (HPC) has changed. Earlier discussed as controversial, surgery and postoperative radiotherapy are now favored as initial procedure by the majority of authors [48, 49]. Again, little is known about the responses in case of unresectable, gross residual, or recurrent tumors. A review by Schier et al covering a period from 1942 to 1987 identified 14 cases of HPC that were treated with radiotherapy alone. There was a fatal outcome in 91% of the cases, mostly around 3 years. Nine cases received no treatment at all: all of them died, 85% after a mean time of 2–3 weeks only [50]. In the experience of the University of Iowa College of Medicine, only one of seven patients initially treated with surgery alone has remained continuously free of disease. In contrast, all four patients treated with surgery and postoperative radiation therapy have remained alive with NED despite the fact that three of these patients had gross residual tumor at the time of irradiation [49]. Altogether, 10 of their 15 patients received radiation therapy at some stage of the management. Local tumor control was achieved at all sites receiving greater than 5500 cGy. Similar results were obtained at the MD Anderson Hospital [48].

In our institution, a recurrent HPC on the meninges (tumor volume about 4–5 ml) disappeared completely after radiotherapy alone with 60 Gy. The patient is alive without evidence of disease 20 years later. Adding razoxane to the radiation treatment has resulted in 3 of 3 patients showing a partial response for a median time of 10 months. Adding razoxane and the vinca alkaloid vindesine to the radiotherapy has given partial responses in 3 of 3 assessable tumors for a median time of 24+ months; 1 additional patient with non measurable residuals at the spine remained recurrence-free 40+ months.

### Chondrosarcomas

Chondrosarcomas (CS) are frequently termed as radioresistant but measurements of the response are rarely presented. Location as a factor of prognosis has to be considered in the judgement of the disease. For instance, chondrosarcomas of the larynx must be seen as an own entity with a favorable prognosis [51, 52]. Likewise, chondrosarcomas of the base of the skull show favorable outcomes if protons or heavy ions were used for irradiation [53, 54]. In contrast to the majority of the literature, however, there are single reports of high local control rates of incompletely resected chondrosarcomas even at the spine and pelvic bones [55]. The authors assumed that the impression of frequent radioresistance is due to a tendency of these tumors to regress slowly and the persistence of skeletal destructions often seen in X-rays. In addition, low radiation doses were used in some earlier reports.

More exact data on the response of chondrosarcomas are found in studies which compared conventional photon irradiation with neutrons. Laramore et al reported an objective response rate (CR + PR) of 33% with photons versus 49% with neutrons [56]. In the experience of McNaney et al only 1 of 7 patients treated with photons alone remained locally controlled [57].

Chondrosarcomas are regarded as not responsive to cytotoxic chemotherapy regardless whether it has been given alone or in combination with radiation treatment. The only report of an improvement of the radioresponsiveness of chondrosarcomas by chemical substances came from England: in 1979 Ryall et al reported a complete and partial response rate of 62% when radiotherapy was combined with the sensitizer razoxane [58].

Stimulated by these observations, we undertook a long-term study to explore the radioresponsiveness of these rare tumors using the protocol of Ryall et al. Between 1984 and 2003, 13 patients with chondrosarcomas were subjected to irradiation together with razoxane. Eight patients had unresectable primaries or recurrences, and 5 received postoperative irradiation (one R-0 and four R-1 resections). The median radiation dose was 60 Gy (range: 54–65 Gy), the median dose of razoxane was 8.5 grams (range: 6–15 g).

Among 8 unresectable or recurrent chondrosarcomas with measurable disease, there were 1 complete and 5 partial regressions, and 2 tumors did not change. The overall response rate was 75%, the median duration of response 23 months. Although 4 of these 8 patients were not locally controlled, the median survival time is not yet reached and lies presently at 45+ months (Table 1). Three of the 4 patients without clear surgical margins remained locally controlled. They survived up to now 12, 21+, 48+, and 160+ months, respectively. One patient with clear margins remained free of disease 87+ months; he had no further follow up. Overall, local control was achieved in 7 of 12 patients who were not radically resected.

Apart from the patients treated with razoxane, 2 other patients received radiation therapy alone by randomization. One of them had no clear margins at surgery, and the other had a recurrence, size 8 × 8 cm. The recurrent tumor progressed even during the radiotherapy. The two patients survived 7 and 9 months, which was the shortest survial of all of our patients with CS.

### Chordomas

Conventional megavoltage irradiation with a median dose of 50 Gy leads to a local control rate of 27% in chordomas [11], while objective tumor regressions are rarely described. For instance, in a recent series of 18 patients with skull base chordomas treated with spot scanning proton beam therapy, no complete or major (> 50%) response was observed [59]. The median time to recurrence or symptoms is 3.5 years in patients with residual disease [60] but sometimes also less [61, 62]. Freedom of symptoms after 10 years was seen in less than 10% of the cases from Zurich [63], and no patient survived 10 years among 4 patients receiving radiotherapy alone in a series of 21 patients of Keisch et al [64]. Therefore, several authors came to the conclusion that inoperable or gross residual chordomas can rarely be cured by conventional irradiation with photons [60, 61, 63]. By using protons or heavy ions, the local control rates rose to around 60% to 70% [6, 59, 65].

We treated 5 consecutive patients with unresectable chordomas at the spine (3 cases) and the base of the skull (2 cases) with radiation and razoxane [66]. The more favorable chondroid variant of chordomas was excluded by a secondary pathology review in all but one case. The median radiation dose was 63 Gy (range: 54–67 Gy); the median total dose of razoxane was 7.6 grams (range: 5.4–11.5 g).

Among 4 measurable tumors there were 2 CR, 1 PR, and 1 NC. All 5 patients survived 5 years and remained locally controlled for 5, 7+, 6.4, 13+, and 15+ years. After a potential median follow-up time of 10 years, 3 of 5 patients are alive without evidence of disease proven by CT and MR imaging. The treatment had little toxicity. Mucosal reactions were predominant as the local side effects, 2 of the 5 patients showed a grade 3 leukopenia (WHO) due to razoxane. So far, no late toxicity at the CNS or the optic nerve was observed.

### Neurogenic sarcomas/malignant schwannomas

Because of the rarity of these tumors there is no data on larger series concerning their radio-responsiveness. Five of our patients with malignant schwannomas received definitive radiation treatment, 2 with radiation alone and 3 with radiation and razoxane. With radiotherapy alone we observed one unchanged and one progressive tumor; with radiotherapy and razoxane there was 1 PR (25 Gy) and 2 unchanged tumors. So far, the radioresponsiveness of neurogenic sarcomas cannot be judged; for the present it seems to be low. This is in accordance with the experience of Raney et al: none of 12 children with gross residual disease survived 3 years; and the authors stress the need for more effective treatment programs especially for those with unresectable tumors [67].

### Miscellaneous other sarcomas

The current knowledge concerning definitive radiotherapy for various rare sarcoma entities such as clear cell, alveolar soft part, or epithelioid sarcomas is restricted to occasional case reports. Therefore, no clear picture is evolving from those data but there is the impression of a rather limited radioresponsiveness. Ferrari et al on behalf of the Italian and German Soft Tissue Sarcoma Cooperative Group reported on 28 pediatric patients with clear cell sarcoma. Eight patients received radiotherapy postoperatively. The only two irradiated patients with gross residual disease after surgery “did not respond significantly.” The authors concluded that radiotherapy may control microscopic residual disease after surgery, chemotherapy is ineffective, and the prognosis is unfavorable for patients with unresectable and large tumors [68]. Shimm and Suit treated 2 epithelioid sarcomas with radiotherapy alone. Both tumors did not change in their size, only 1 was locally controlled, and both cases developed distant metastases [69]. A partial remission and an unchanged tumor was observed in two of our own cases with alveolar soft part sarcomas treated with radiotherapy and razoxane. Both cases remained locally controlled after radiation doses of 40 and 30 Gy but died from distant metastases after 9 months and 6 years.

## DISCUSSION

The terms radioresponsive and radiosensitive are synonymous in everyday usage. It will be difficult to associate objective responses exclusively with “radioresponsiveness” and the degree of tumor necrosis with the generic term “radiosensitivity”. In reporting on definitive radiotherapy, it should however be sufficient to describe the quality and degree of objective responses and their duration, local control, and survival. Those data would speak for themselves and cover radiosensitivity (histopathologic necrosis) and radioresponsiveness (shrinking of a mass) as well. Knowledge about the radioresponsiveness of sarcomas is of value for treatment decisions, for example, a good response may more likely permit the conservative surgical excision of tumors of borderline resectability, or for having data to be compared with results from novel treatments.

The radioresponsiveness of sarcomas as a group is moderate at best. About 40%–50% of the tumors show transient objective responses to radiotherapy alone; complete responses are less frequent. Some reports deal with histopathologic response rates, that is, the degree of tumor necrosis [12, 13, 70, 71], but the prognostic significance of a 60% to 90% tumor necrosis, values most frequently observed, remains unclear. As mentioned, several measures led to an increase of local control and objective responses in sarcomas. Interestingly, the use of contemporary cytotoxic chemotherapy represented by doxorubicin-based regimens together with concomitant radiotherapy seems to be of low or modest efficacy. A study with neoadjuvant doxorubicin-based chemo-radiation in STS revealed a tumor-downstaging in 18% of patients only although there was a survival gain [72]. Another work reported no radiographic partial response or disease progression among 27 patients but there were some histologic responses in the series [71]. The amount of necrosis was dependent on the doxorubicin dose given together with radiotherapy. Eilber et al reported on neoadjuvant combined treatments for high-grade extremity STS. Major necrosis increased to 48% with the addition of ifosfamide as compared to 13% of the patients in all other protocols combined [12]. In a study using preoperative radiotherapy together with the MAID regime (MAID = mesna, adriamycin, ifosfamide, dacarbazine), DeLaney et al saw no clinical complete responses and 10.6% partial responses among 48 patients; 36 patients (75%) did not respond [70]. Six patients (13%) had radiographic evidence of “progression” by tumor measurements on the MRI scans. Most of the tumors in these 6 patients, however, showed pathologically confirmed necrosis of various degree. The authors suggested that the radiographic progression represented swelling of tumor secondary to the osmotic effect of necrosis [70].

This overview indicates that the radiation response of sarcomas is dependent on the histopathology and the addition of chemical modifiers of the radiation response. Radiotherapy alone leads to major but transient responses in about 50% of lipo-, fibro-, leiomyo-, or chondrosarcomas. The response rate is less than 50% in malignant fibrous histiocytomas, synovial sarcomas, neurogenic sarcomas, and other rare entities. No definite conclusions are allowed for the latter forms since the available data are sparse. The response rates may be increased to 75% by the use of chemical modifiers. In particular, angiosarcomas showed promising responses if biologicals, angiomodulating, and/or tubulin affinic substances are given together with radiation therapy.

The radiosensitizing agent razoxane is able to increase the duration and quality of responses in fibro-, lipo- and leiomyosarcomas compared to irradiation alone. Even in difficult-to-treat tumors such as chondrosarcomas or chordomas, photon irradiation together with razoxane induces objective responses in the majority of patients. Compared to data from the literature, this treatment combination offers an alternative to high-LET irradiation. Although the small number of chordomas precludes far-reaching conclusions, the fact of nonselected cases, the longer follow-up, and the favorable responses would make it worthwhile to test razoxane together with or against a radiation therapy with protons or heavy ions.

Razoxane is a largely neglected radiosensitizer which has an interesting spectrum of modes of action. It is of value in the treatment of STS because of its proven radiosensitizing effects [9, 16, 73] and its potential to normalize pathologic tumor blood vessels [35, 74, 75]. This angiometamorphic mode of action commends the drug especially for its use in angiosarcomas. In addition, the drug induced a growth rate slowdown of transplanted tumors [76], and the development of distant metastases to the lungs was completely suppressed in preclinical trials [74, 75, 77]. Razoxane is also antiinvasive [33], and it inhibits the topoisomerase II [78]. Unfortunately, since 2004 razoxane has been discontinued by Cambridge Laboratories (UK). It is to be hoped that another pharmaceutical company will shortly take up again the unique possibilities of the drug. Meanwhile, the use of dexrazoxane could be a reasonable alternative. From our experience with radiotherapy plus razoxane in STS, it would seem to be mandatory in any phase III trial of a new combination to use radiation and razoxane as the control arm.

This report may represent a first inventory of radiation responses in subunits of sarcomas. It might be of value to those who intend to analyze new radiosensitizers or novel combination chemotherapies in conjunction with radiation treatment. Simple pilot trials with new drugs are needed since the rates of complete sustained regressions in different sarcomas achieved by the concomitant use of chemical modifiers and irradiation are still far from being satisfying.

## Figures and Tables

**Table 1:** Radiotherapy and razoxane in 8 patients with unresectable primary or recurrent chondrosarcomas.

#	Age/gender	Definitive irradiated tumors			Results of radiotherapy + Rz				Remarks
		Primary	Size (cm)	Grade	Response	Duration	Local	Survival	
						(months)	control	(months)	

1	61/m	Os ileum	20 × 15	G1	PR	20	No	38	Died from local recurrence
2	58/m	Larynx	5 × 4	G2	PR	85	Yes	85	Intercurrent death
3	74/f	Groin	17 × 11	G3	PR	22	Yes	22	Lung metastasis
4	80/f	Knee	15 × 14	G2	PR	90	Yes+	90	+ Sekund amputation
5	24/f	Orbita	0.8 × 0.8	G2	CR	48	No	56+	Salvage surgery after 48 months
6	65/m	Sternum	3 × 2	G1	NC	25	No	52+	Salvage surgery after 25 months
7	44/f	2nd rib	8 × 8	G2	NC	22	Yes∗	22	∗ No follow-up last 5 months
8	81/m	Humerus	6 × 5, 4 × 3	G2	PR	16	No	28+	Regrowth after 16 months
